# Analog Memristive Synapse in Spiking Networks Implementing Unsupervised Learning

**DOI:** 10.3389/fnins.2016.00482

**Published:** 2016-10-25

**Authors:** Erika Covi, Stefano Brivio, Alexander Serb, Themis Prodromakis, Marco Fanciulli, Sabina Spiga

**Affiliations:** ^1^Laboratorio MDM, Istituto per la Microelettronica e i Microsistemi - Consiglio Nazionale delle Ricerch (CNR)Agrate Brianza, Italy; ^2^Nano Group, Department of Electronics and Computer Science, University of SouthamptonUK; ^3^Dipartimento di Scienza Dei Materiali, Università di Milano BicoccaMilano, MI, Italy

**Keywords:** memristor, resistive switching, HfO_2_, artificial synapse, synaptic plasticity, spike time dependent plasticity, spiking neuromorphic network, unsupervised learning

## Abstract

Emerging brain-inspired architectures call for devices that can emulate the functionality of biological synapses in order to implement new efficient computational schemes able to solve ill-posed problems. Various devices and solutions are still under investigation and, in this respect, a challenge is opened to the researchers in the field. Indeed, the optimal candidate is a device able to reproduce the complete functionality of a synapse, i.e., the typical synaptic process underlying learning in biological systems (activity-dependent synaptic plasticity). This implies a device able to change its resistance (synaptic strength, or weight) upon proper electrical stimuli (synaptic activity) and showing several stable resistive states throughout its dynamic range (analog behavior). Moreover, it should be able to perform spike timing dependent plasticity (STDP), an associative homosynaptic plasticity learning rule based on the delay time between the two firing neurons the synapse is connected to. This rule is a fundamental learning protocol in state-of-art networks, because it allows unsupervised learning. Notwithstanding this fact, STDP-based unsupervised learning has been proposed several times mainly for binary synapses rather than multilevel synapses composed of many binary memristors. This paper proposes an HfO_2_-based analog memristor as a synaptic element which performs STDP within a small spiking neuromorphic network operating unsupervised learning for character recognition. The trained network is able to recognize five characters even in case incomplete or noisy images are displayed and it is robust to a device-to-device variability of up to ±30%.

## 1. Introduction

The human brain is a massively parallel, fault-tolerant, adaptive system integrating storage and computation (Kuzum et al., 2013; Matveyev et al., 2015). Moreover, it is able to visually recognize a large amount of living beings and objects and to process huge volumes of data in real-time (Kuzum et al., 2013; Yu et al., 2013a; Wang et al., 2015). Therefore, biologically-inspired systems are attracting a lot of interest as vehicles toward the implementation of real-time adaptive systems for a variety of applications. In such applications, the system is required to continuously adapt to time-varying external stimuli in an autonomous way, therefore an on-line learning without external supervision is preferable (Serb et al., 2016). In neuromorphic hardware, learning is obtained through reconfiguration of the connectivity of a network through local modulation of synaptic weights. The adjustment of the weight of a single synapse, i.e., plasticity, should follow simple update rules that can be implemented uniformly across the entire network and allow unsupervised learning. In this respect, spike timing dependent plasticity (STDP) has been recognized as one of most promising, because it establishes that the weight of a synapse is adjusted according to the timing of the spikes fired by connected neurons (Serrano-Gotarredona et al., 2013; Bill and Legenstein, 2014; Ambrogio et al., 2016b).

Recently, the implementation of artificial synapses with memristor devices has been proposed. Memristors (memory + resistor) are compact two terminal devices that change their resistance when subjected to voltage stimulation. The memristor resistance state can be considered inversely proportional to the synaptic weight. Various practical implementations have been proposed, such as phase change (Kuzum et al., 2012; Ambrogio et al., 2016b), ferroelectric (Du et al., 2015; Nishitani et al., 2015), spin transfer torque (Querlioz et al., 2015) devices, and oxide-based resistive switching memristors (Wang et al., 2015; Ambrogio et al., 2016a). When memristors are employed in neuromorphic networks, two main operational modes are used, binary and analog. The former relies on memristors featuring only two states, high resistance state (HRS) or low resistance state (LRS), and it is proved to be effective in specific applications (Suri et al., 2013; Wang et al., 2015; Ambrogio et al., 2016a). On the other hand, analog evolution of device resistance is desirable to improve the robustness of the network (Bill and Legenstein, 2014; Garbin et al., 2015; Park et al., 2015), but the difficulty of operating memristors in an analog fashion renders hardware implementations of networks with analog synapses still challenging (Garbin et al., 2015). Indeed, several memristors show only a partial analog behavior, either when increasing the resistance (synaptic depression), which is common in filamentary devices as oxide-based memristors (Kuzum et al., 2013; Yu et al., 2013a), or when decreasing the resistance (synaptic potentiation) as in some kinds of phase change memristors (Eryilmaz et al., 2014). Well established protocols to obtain analog behavior require controlling of the current flow through the memristor (Yu et al., 2011; Ambrogio et al., 2013), or the modulation of either the time width (Park et al., 2013; Mandal et al., 2014) or the voltage (Kuzum et al., 2012; Park et al., 2013) of the spike. However, this device programming requires the use of extra circuit elements for monitoring the state of the memristor and shaping the spike accordingly. A second proposed approach is to consider multi-memristor synapses (compound synapse with stochastic programming) (Bill and Legenstein, 2014; Burr et al., 2015; Garbin et al., 2015; Prezioso et al., 2015) at the expense of increased area consumption. Only recently some works demonstrated analog behavior in both potentiation and depression without current or voltage control (Park et al., 2013; Covi et al., 2015, 2016; Matveyev et al., 2015; Brivio et al., 2016; Serb et al., 2016).

Within this class of devices, unsupervised learning based on STDP has been successfully demonstrated and analyzed in detail for binary synapses or compound synapses (with binary memristors) (Suri et al., 2013; Bill and Legenstein, 2014; Ambrogio et al., 2016a,b). Some works deal with networks utilizing analog resistance transition in only one direction, either in depression (Yu et al., 2013b) or in potentiation (Eryilmaz et al., 2014). Only few works use analog synapses to simulate neuromorphic networks, as an example Querlioz et al. (2013), Yu et al. (2015), and Serb et al. (2016). The latter, in particular, proposes a network realized in part with real hardware analog memristors and in part with software simulation.

In this framework, we propose a fully analog oxide-filamentary device as a memristive synapse for networks with deterministic neurons implementing unsupervised learning. The proposed memristor features an analog modulation of its resistance in various long-term functional plasticity spiking conditions and it emulates a type of homosynaptic STDP learning rule. To prove its usefulness in deterministic STDP-based networks, a simple fully-connected spiking neuromorphic network (SNN) for pattern recognition is conceived and simulated. The SNN consists of 30 neurons (25 pre-neurons disposed in a 5 × 5 layer and 5 post-neurons) and 125 synapses. The network is trained with an associative unsupervised STDP-based learning protocol. After training, the SNN is able to recognize five characters displayed as 5 × 5 black-and-white pixels images even when incomplete characters or noisy ones (intended as purely additive noise) are displayed. Moreover, the SNN is proved to be robust against device-to-device variability.

## 2. Materials and methods

The device stack is made of 40 nm TiN/5 nm HfO_2_/10 nm Ti/40 nm TiN layers and the area of the device is 40 × 40 μm^2^. Ti and TiN layers are deposited by magnetron sputtering and the HfO_2_ layer is deposited by atomic layer deposition at 300 °C, as described elsewhere (Brivio et al., 2015; Frascaroli et al., 2015). The switching mechanism of the proposed memristor is filamentary (Brivio et al., 2014), i.e., it is based on the disruption and the restoration of a conductive filament formed inside the oxide.

**Figure 1:**
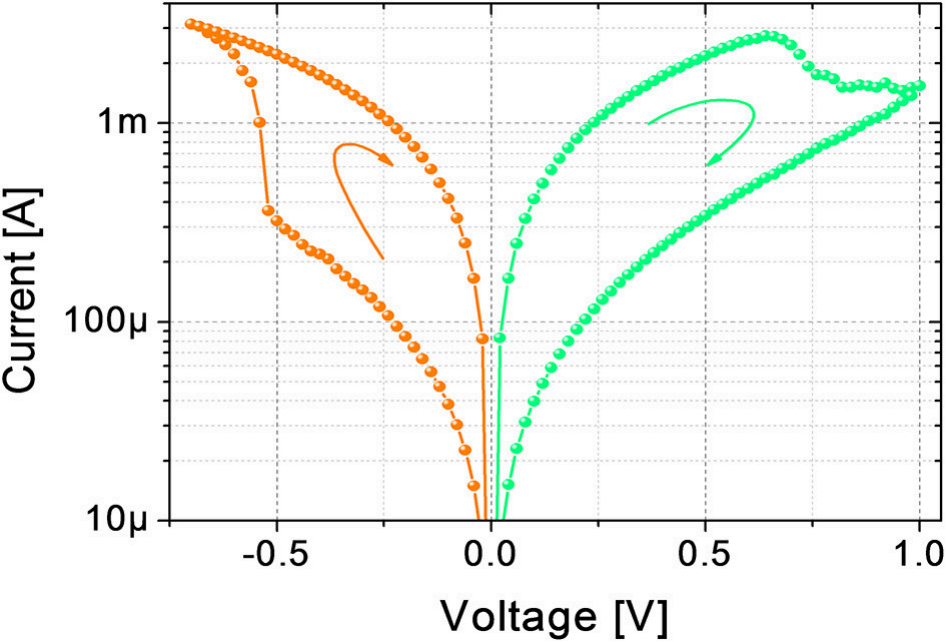
**DC characterization of the device**. Transition from LRS to HRS is obtained with a DC sweep from 0 V to 1 V, transition from HRS to LRS is obtained with a DC sweep from 0 V to −0.7 V.

The electrical DC characterizations are performed using Source Measuring Units (B1511B and B1517A) of a B1500A Semiconductor Device Parameter Analyzer by Keysight. Figure 1 shows a typical I-V curve of the device. In its pristine state, the device has a conductance of tens of nS (not shown). A forming operation (DC current sweep up to 150 μA) at around 1.8 V (data not shown) is needed to bring the device in its LRS for the first time. To switch the device from LRS to HRS, and vice versa, DC sweeps from 0 V to 1 V (LRS to HRS) and from 0 V to −0.7 V (HRS to LRS) are applied. The device maximum resistance (read at 100 mV) ratio obtainable in DC is about one order of magnitude, which is in agreement with the literature (Garbin et al., 2015; Matveyev et al., 2015; Wang et al., 2015).

The device response to spike stimulation has been characterized either by trains of pulses with increasing amplitude and fixed time width or by repetition of the same spike. In the former case, during depression spike amplitude ranges from 0.1 to 1.2 V, during potentiation, from −0.1 to −0.65 V. The same spike is repeated 5 times before the amplitude is incremented by 50 mV (decremented by −50 mV for negative voltages) and the pulse duration is fixed at 100 μs. Measurements are performed using the custom instrument described in Berdan et al. (2015). In the second experiment, the trains of identical pulses are constituted by 300 repetitions of −550 mV—high and 25 μs—long pulses for potentiation and 300 repetitions of 700 mV—high and 20 μs—long pulses for depression. This second pulse scheme is implemented by a custom setup interfacing High Voltage Semiconductor Pulse Generator Unit (B1525A) and Source Measuring Units of a B1500A. The motivation for the choice of the spike parameters will be given in Section 3. In both experimental procedures, reading operation is carried out using a voltage amplitude which induced no changes in the device resistance.

STDP experiments are carried out placing the device between two spiking channels, i.e., two Waveform Generator/Fast Measurement Units (B1530A) of the already mentioned B1500A, acting as spiking neurons. The relative timing between the two overlapping spikes from the two neurons is mapped in a voltage amplitude, as it will be described in Section 3.2.

The SNN is developed and simulated in Matlab® environment. The network is a simple fully connected winner-take-all SNN of 30 integrate-and-fire neurons, of which 25 are pre-neurons and 5 post-neurons. The pre-neurons are arranged in a 5 × 5 layer and each pre-neuron is connected to all the post-neurons through 125 artificial synapses. The learning method is unsupervised and the experimental STDP data used to update the synaptic weights during learning are collected in a look up table. The operating principle of the network will be described in detail in Section 3.3. Using the same Matlab® software, a graphic user interface (GUI) is developed to enhance the software usability (further details in the Supplementary Figure 1).

## 3. Results

The tests described in the following are carried out in order to provide a thorough overview of the device behavior which is finally exploited in a simple example of neuromorphic computation. The present section is therefore divided in three parts. In the first one, long-term functional plasticity is investigated through two different spiking algorithms, which are exploited to achieve a form of STDP learning rule, in the second part. Finally, a SNN is presented.

### 3.1. Long-term functional synaptic plasticity

The plasticity of the device is investigated through two different spiking stimulations, which are fundamental to achieve the shape of STDP required in learning.

**Figure 2:**
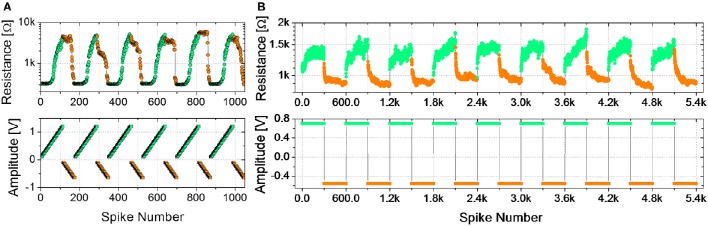
**(A)** Potentiation (orange) and depression (green) cycles using ramped trains of spikes. Spike time width 100 μs, 5 repetitions. Potentiation: ramps from −0.1 V to −0.65 V. Depression: ramps from 0.1 V to 1.2 V. Upper graph: device resistance after spikes; lower graph: spike amplitude. **(B)** Potentiation (orange) and depression (green) cycles using trains of 300 identical spikes. Potentiation: spike amplitude −0.55 V, time width 25 μs. Depression: spike amplitude 0.7 V, time width 20 μs. Upper graph: device resistance after spikes; lower graph: spike amplitude.

Figure 2A shows the evolution of the device resistance during some potentiation and depression cycles (top panel) using trains of spikes of fixed time width and increasing amplitude (bottom panel). The maximum voltages for potentiation (−650 mV) and depression (1.2 V) are those leading to a maximum resistance change of about one order of magnitude and are close to the maximum voltages used in DC operation (Figure 1). During depression (resistance increase, green circles), the first spikes, corresponding to lower voltages (see bottom panel of Figure 2A), do not induce any resistance change up to a voltage threshold which can be identified at about 550 mV. As the threshold is overcome, the resistance starts increasing gradually. The device therefore presents several intermediate resistive states throughout the programming window. Similarly, during potentiation (resistance decrease, orange circles), several intermediate states are reached between the maximum and minimum resistances using spikes with increasing voltage amplitude. It can be noted that, in this case, the resistance change begins at different voltage levels from cycle to cycle, but for voltages higher than −500 mV a resistance decrease can always be observed. Therefore, −500 mV is considered the voltage threshold for potentiation. It is worth noticing that time widths, as well as voltages, influence the resistance evolution, as already reported by Covi et al. (2015) for similar devices. On the other hand, resistance changes are more sensitive to voltage variations rather than to time widths variations, so that for time widths in the range of 10 to 100 μs roughly the same voltages can be applied for obtaining the same resistance evolution. It has to be mentioned that a stair-case like algorithm, like the one used here, is not practical to implement in real large-scale system, because requires neurons to keep track of previous activity. On the other hand, the testing procedure reported in Figure 2A is useful for characterizing the device and to clarify the functioning principle of the STDP implementation described below, which has actually been proposed as a learning rule for practical implementation of neuromorphic hardware (Saïghi et al., 2015).

In the set of measurements shown in Figure 2B, plasticity is investigated as a function of trains of identical spikes. Some depression/potentiation cycles are performed. During both potentiation and depression, the resistance gradually changes, featuring several intermediate states between the LRS and the HRS. In all the cycles, the resistance rate change is not constant with respect to the number of spikes. Indeed, for both potentiation and depression the resistance change is faster for the first spikes. In general, analog resistance variation due to trains of identical spikes can be found for voltages values close to the voltage thresholds, identified by the results of voltage staircase stimulation for similar time widths (as that shown in Figure 2A). Indeed, gradual resistance change is achievable as an intermediate regime between a low voltage stimulation, which does not affect the resistance, and a high voltage stimulation, which induces a digital behavior (Covi et al., 2015). The resistance window obtained through identical pulses is in the order of 2, which has been considered sufficient when dealing with neuromorphic systems (Kuzum et al., 2012; Prezioso et al., 2016).

### 3.2. Homosynaptic input-specific plasticity toward learning

**Figure 3:**
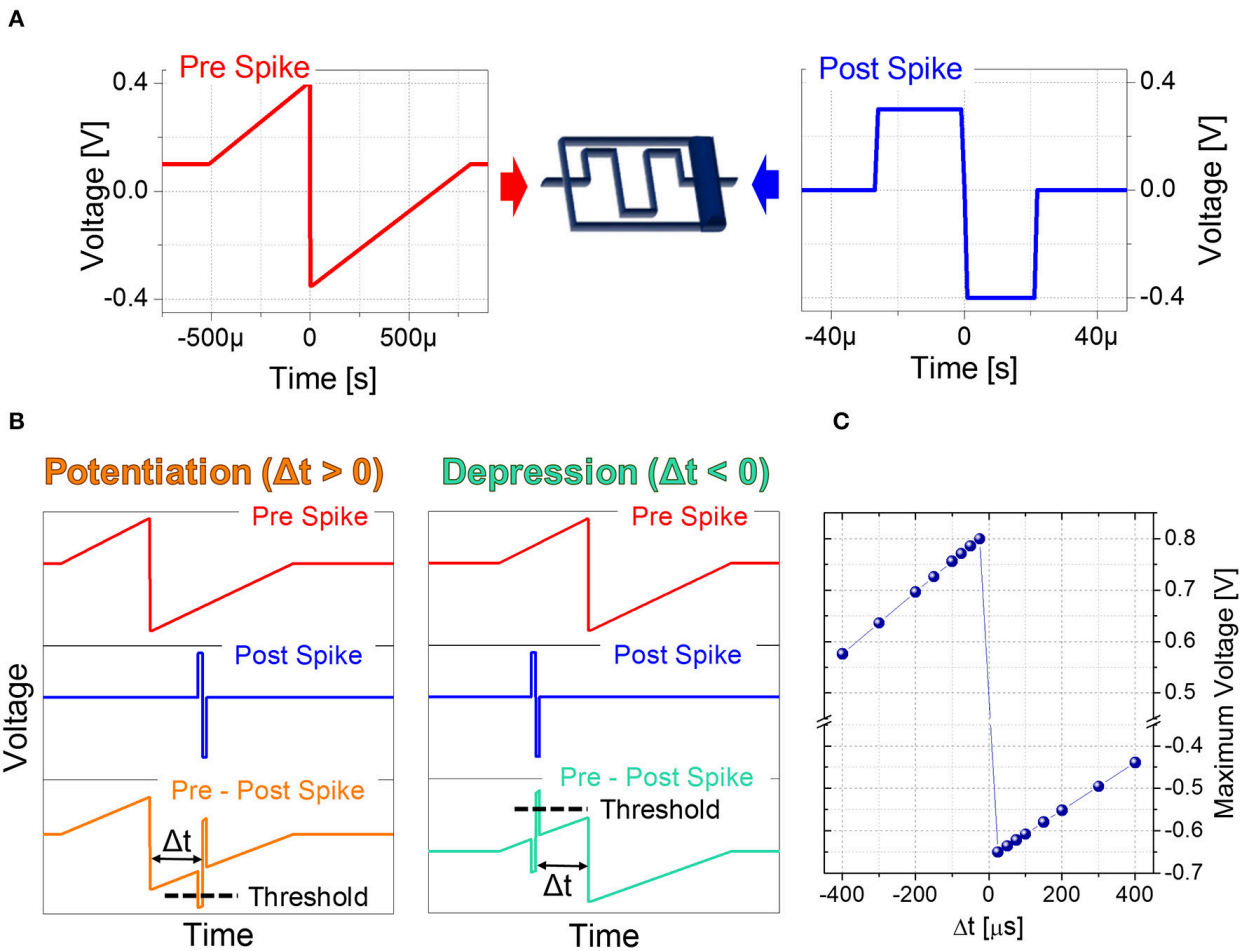
**(A)** Setup for Spike Time Dependent Plasticity and waveforms used as pre-spike (left) and post-spike (right) in STDP experiments. **(B)** Overlapping of pre-spike and post-spike to obtain a potentiation (left) and a depression (right). **(C)** Voltage-to-delay time mapping. Resulting voltage across the artificial synapse as a function of Δt.

Based on the plasticity results described in Section 3.1 as a function of voltage modulation and spike repetition, STDP experiments relying on engineering of pre- and post-spike superimposition are carried out. Indeed the voltage drop on the memristor is modulated according to the voltage difference resulting from the superimposition of pre- and post-spike waveforms, which depends on their relative timing. To this aim, pre-spike is shaped as a triangular-like pulse (Figure 3A), thus acting as a bias performing the voltage-to-time mapping. The rectangular-like shape of the post-spike (Figure 3A) determines the supra threshold spike width. Figure 3B reports two examples of the superimposition of pre- and post-spikes giving either potentiation or depression and Figure 3C reports the quantitative voltage-to-delay-time mapping. In particular, the resulting maximum voltage dropping on the device depends on Δt and varies between −650 mV for potentiation and 800 mV for depression.

**Figure 4:**
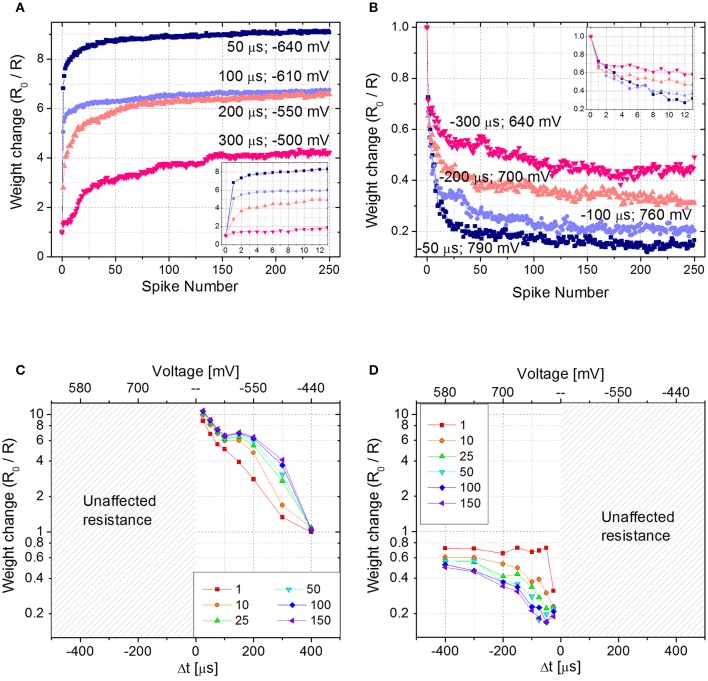
**(A)** Potentiation and **(B)** depression dynamics with 250 identical spikes. Different voltage amplitudes and delay times are explored. The values of both voltage amplitude and Δt are written nearby each curve. Insets: detail of the first 12 spikes. **(C,D)** Spike Time Dependent Plasticity learning curve for different number of pre- post-spikes pair repetitions (Δt > 0 and Δt < 0). *R*_0_ is the initial HRS **(C)** and LRS **(D)**.

To emulate STDP with Δt > 0 (Δt < 0), first the device is brought in its HRS (LRS) with a DC sweep, then 250 identical pairs of pre- and post-spikes are applied to the top and bottom electrodes of the device, respectively, keeping Δt constant. The experiment is repeated for different delay times (Δt) and each time the parameter Δt is varied, the device is reinitialized accordingly. Figures 4A,B show the device resistance evolution as a function of spike pair repetitions for different delay times in both potentiation (Figure 4A) and depression (Figure 4B). During potentiation and for every delay time, resistance decreases quickly in the initial phase (about ~25 repetitions) before slowing down markedly in later phases (please notice the vertical scale as going like *R*_0_/*R* with the increase of the number of spikes, in qualitatively agreement with Figure 2B). The same qualitative trend is respected also during depression (Figure 4B): the first 10–20 spike pair repetitions significantly change the resistance, whereas the following ones are less effective, until a saturation level is reached after ~150–200 spikes. In both potentiation and depression, the variation of Δt, i.e., the voltage drop, drives the amplitude of the resistance change, i.e., the longer the delay time, the lower the change in resistance. Moreover, Δt affects the resistance change rate in the initial stage of the plasticity operation, i.e., the smaller the delay time (i.e., the higher the voltage drop), the sharper the resistance evolution (e.g., compare the blue and pink curves of Figures 4A,B).

Figures 4C,D show the STDP curve represented as the normalized resistance change as a function of the spike delay (and consequently of the voltage amplitude, as shown in the top x-axis of Figures 4C,D) for few representative fixed numbers of spike pair repetitions (1, 10, 25, 50, 100, and 150). The plots, which are derived from aforementioned results, qualitatively follow the biological STDP curve shown in Bi and Poo (1998). In accordance with Figures 4A,C shows that when Δt is positive and small, the first spike pair induces a resistance variation equal to 75% of the dynamic range. As a consequence, the following repetitions have a reduced effect in further changing the device resistance. On the contrary, when Δt is longer and the resulting spike voltage amplitude is lower, the spike repetitions play an important role in the evolution of the device resistance. Indeed, it becomes progressively more pronounced with increasing Δt. This effect is valid up to a point where Δt is so large that the voltage drop across the device does not exceed the device threshold and no more changes in device resistance are induced, regardless of the number of applied spikes. The same effect is shown also in Figure 4D, where results for negative Δt are plotted, even though here the effect is less pronounced. Indeed, a change in the synaptic weight is present also for Δt = −400 μs. This result is in agreement both with Figure 2A, where the effect of the voltage amplitude on the device resistance is shown, and with Figure 2B, where it is demonstrated that the weight change progressively decreases with increasing spike repetition number.

It is worth mentioning that when the device behavior is tested for Δt > 0 (Δt < 0), the device is first brought in its HRS (LRS). In case the memristor in the LRS (HRS) is subjected to pulses with Δt > 0 (Δt < 0), no changes in its resistance would occur, since the synapse is already completely potentiated (depressed). This is explicitly shown in Figures 4C,D, where for negative (positive) delay times no resistance changes are shown.

From Figures 4C,D, a behavioral difference between potentiation and depression dynamics emerges. Despite in both cases the final resistance is strongly influenced by the applied voltage amplitude, during potentiation the applied voltage affects the change in the device resistance starting from the very first spike pair, whereas during depression the effect of the voltage is more evident from the second spike pair on, rather than in the first. Such asymmetry of the curve, even though in principle improvable by optimizing the spike shapes, does not affect the possibility of using the STDP rule for a neuromorphic network.

### 3.3. Associative unsupervised learning in spiking neuromorphic networks

The goal of the following Section is to demonstrate the operation of a small unsupervised network which makes use of the plastic response of the memristor described above to emulate the functionality of a synapse. To this end, we concentrate just on a network with fixed timings, i.e., restricting for simplicity to a subset of the STDP data presented in Section 3.2. More specifically, the curve with Δt = 300 μs of Figure 4A is selected for potentiation and the one with Δt = −50 μs of Figure 4B for depression. Of course, the shape of the STDP curve provides additional degrees of freedom that can be exploited for addressing more biologically plausible learning algorithms, e.g., for the treatment of gray-scale or color images. However, such applications go beyond the scope of the present manuscript.

**Figure 5:**
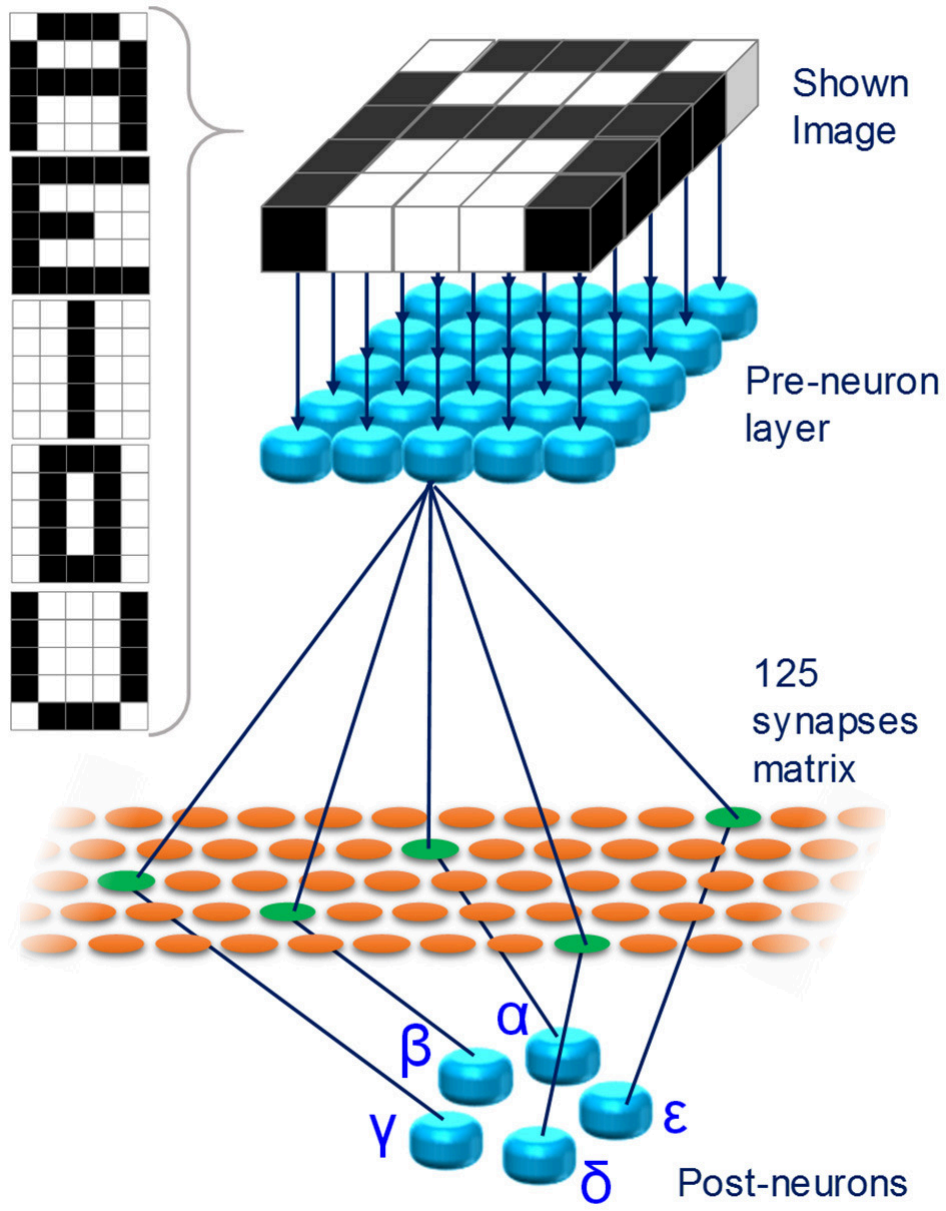
**Proposed fully connected SNN**. 25 pre-neurons are connected to 5 post-neurons through a layer of 125 artificial synapses. Each pixel of the images shown to the network are associated with a pre-neuron. Inset: images showed to the SNN during the training phase.

Figure 5 shows the proposed SNN. For ease of visualization, in Figure 5 only a limited number of the connections between pre- and post-neurons is shown. Each of the 25 pixels composing the images is associated to a different pre-neuron. Initially, the network is untrained and a learning phase is executed. At the end, the SNN is able to recognize 5, capital characters (*A, E, I, O*, and *U*, Figure 5, inset) given as 5 × 5 pixel black-and-white images. The network learns through an unsupervised STDP protocol. Once the training session is over, the network is able to recognize incomplete or noisy images, representing any of the characters, following a winner-take-all approach.

**Figure 6:**
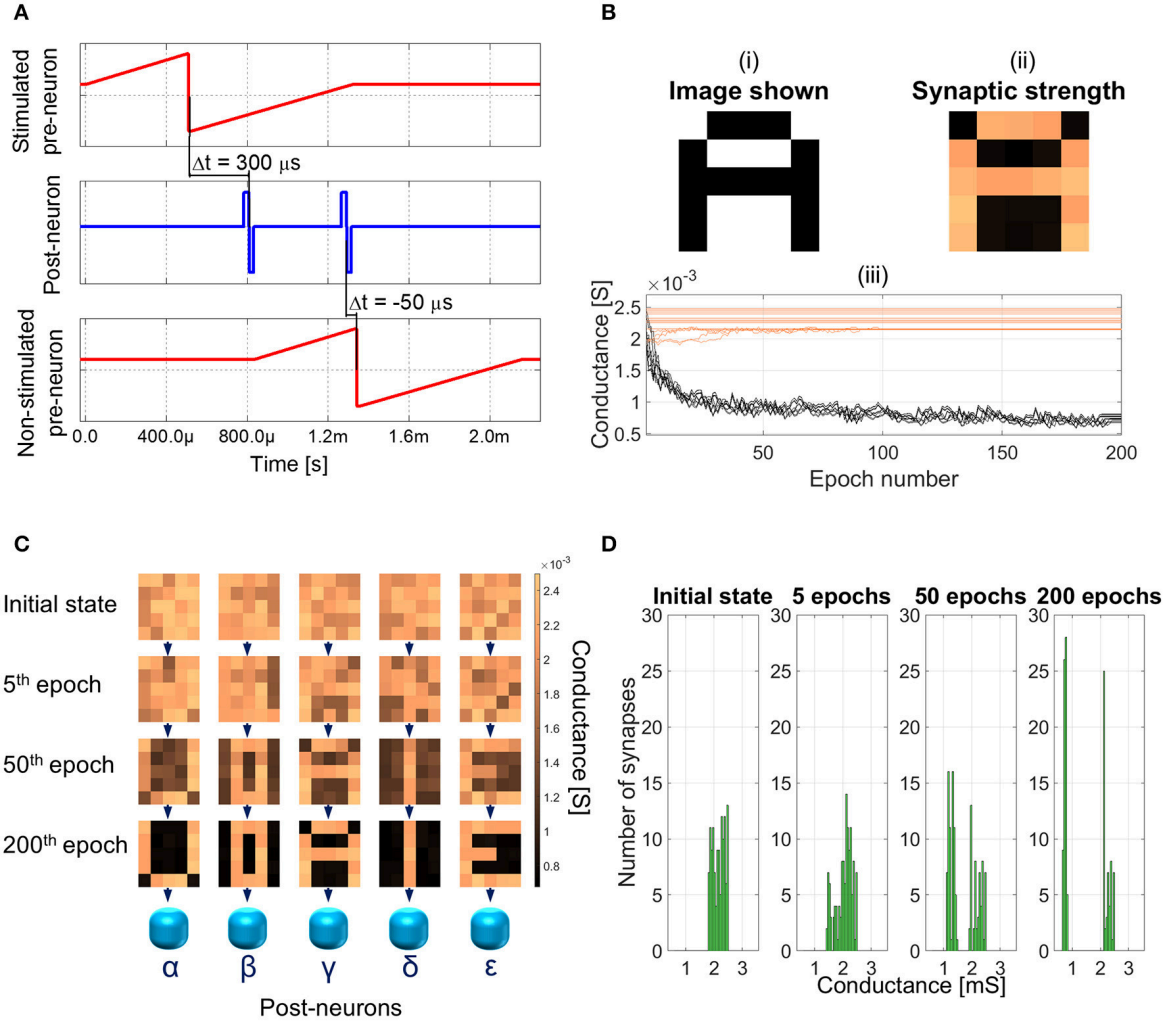
**(A)** Training session: spiking diagram of one epoch. The training character is shown at 0 s and the duration of an epoch is about 2.15 ms. **(B)** Image shown to the network (top left panel), synaptic weights after 200 epochs (top right panel), and detailed synaptic weight evolution during training session of character *A* (bottom panel). Black lines represent the synapses which are being depressed during the session and orange lines the ones potentiated. **(C)** Example of synaptic weight changes during a learning session. Each 5 × 5 matrix represents the group of 25 synapses contributing to the firing of neurons α to ϵ. Color bar on the right indicates the conductance range of the synapses. Increasing the number of epochs (from top to bottom), the SNN specializes each post-neuron to recognize a different character. **(D)** Distribution of the synaptic weights during the training session.

The plasticity of the memristor plays most of the role in the learning session of the SNN. The training is performed one character at a time. As an example, the procedure to make the network learn letter *A* is described. The same procedure is then used for all the other characters. The spiking diagram of the neurons is shown in Figure 6A and it will be explained together with the unsupervised learning protocol.

At first, character *A* is shown to the network. Black pixels stimulate the associated pre-neurons (Figure 5), which fire toward all the post-neurons (Figure 6A, top panel). Post-neurons integrate the signals and the one which first reaches its threshold voltage (e.g., post-neuron γ), which is fixed and equal for all the post-neurons, fires back to all the pre-neurons (Figure 6A, middle panel). The fired spike has three effects: (i) the discharge of all the other post-neurons, following the winner-take-all rule; (ii) the potentiation of the synapses connecting pre-neurons associated with black pixels and post-neuron γ (Δt > 0); (iii) the triggering of the firing of the pre-neurons associated with white pixels (Figure 6A, bottom panel). Afterward, about 500 μs after the first spike, post-neuron γ fires again (Figure 6A, middle panel), thus depressing the synapses connecting it with firing pre-neurons (Δt < 0). Pre-neurons associated with black pixels are in their absolute refractory period, therefore the second spike form post-neuron γ has no effect on them. This procedure of neurons handshaking, lasting about 2.15 ms, is called epoch and it occurs each time an image is presented to the SNN during training session. To reach successful learning (i.e., each post-neuron is specialized for a different character) with a probability of 99%, the same character is shown to the network up to 200 times (epochs).

Figure 6B shows an example of training session for letter *A*. The Figure is an excerpt extracted from the video VideoS1.mp4, which can be found in the Supplementary Material and it summarizes the whole 200 epochs occurred to specialize the SNN to recognize character *A*. In panel (i), the image shown to the network is represented. In panel (ii), the synaptic weight after 200 epochs of the subset of synapses contributing to the firing of post-neuron γ is shown. The potentiated synapses are the orange squares in the panel, whereas the depressed ones are colored in black. A close correspondence of panels (i) and (ii) is evident, which is at the basis of the relationship between potentiated synapses and character learned. In panel (iii), the weight evolution as a function of the number of epochs is shown. The depressed synapses (black lines) tend to converge to the lowest conductance value of about 800 μS. On the contrary, the potentiated synapses (orange lines) show a very slight change in the conductance, if any, due to the limit imposed by the initial condition of the synaptic layer. Indeed, the initial conductance of each synapse is set in the range from 1.8 to 2.5 mS. The initial distribution is the result of a potentiation operation and it simulates the device-to-device variability plausible in a real network. Both the width and the average value of the initial weight distribution are fundamental to allow the SNN to uniquely specialize post-neurons during learning session. The variability in the initial resistance, which is actually unavoidable for real devices, allows one post-neuron to be favored with respect to the others and therefore to fire first. The narrower the distribution of initial synaptic weight toward high conductance values, the higher the probability of success during learning. This is true up to the unrealistic situation where all the synapses have the same weight and, therefore, all post-neurons would fire simultaneously, thus failing the learning task. Similarly, the widening of the initial state range leads to a situation where two similar characters, e.g., *E* and *U*, fall in the basin of attraction of the same post-neuron, thus resulting in an unsuccessful learning (i.e., the SNN forgetting the former character and specializing the same post-neuron to recognize the latest character presented). The same erroneous behavior is obtained if the average value of the initial distribution is moved toward lower conductances.

An example of complete training session is illustrated in Figure 6C (an animation of the first 50 epochs is shown in Supplementary Material, VideoS2.mp4). Each 5 × 5 matrix in Figure 6C represents the group of 25 synapses contributing to the firing of post-neurons α to ϵ. Initially, all the weights are randomly distributed between 1.8 and 2.5 mS. Increasing the number of epochs (in the Figure, initial state, 5th, 50th, and 200th epochs are shown), the weight of each synapse gradually changes until, at the 200th epoch, the SNN is trained and the characters are recognizable also in the synaptic layer. In addition, Figure 6D evidences the distribution of all the 125 synaptic weights in the initial states and after 5, 50, and 200 epochs. It can be noted that during the session the initial distribution, which is initially grouped unimodally toward the highest conductive values, is split in two, one group for depressed synapses and one for potentiated ones, which is consistent with the results shown in Figure 6B, panel (iii).

Similar to the training session, during recognition, when an image is shown to the SNN, the stimulated pre-neurons fire toward all the post-neurons. The post-neuron which is first charged above its threshold fires, both recognizing the character shown and discharging the other post-neurons.

The recognition tests are carried out on 100 networks configurations resulting from the same number of learning simulations with different initial synaptic weights. The test set can be divided into two classes of images, one with missing pixels and one with additive noise (Supplementary Figure 2). In the first test, several images with missing black pixels are shown to the SNN. The results demonstrate that in the worst case the network is always (100% recognition rate) able to recognize the character if the percentage of missing pixel is equal to or lower than 21% for character *A*, 27% for character *E*, 20% for character *I*, 33% for character *O*, and 18% for character *U*. In the second test, noisy images are shown to the network. The test images are chosen among the ones considered mostly critical for the SNN to be recognized, so that worst cases could be explored. Further details about the images shown and the choice criterion can be found in the Supplementary Figures 2, 3, and Supplementary Table 1. The network recognition rate resulted 85.71% for images with up to 4 noise pixels. However, the recognition rate is correlated with the number of epochs in the training session. As already mentioned, a training session for a character consists of 200 epochs and it almost always leads to a successful learning. If the number of epochs during training is reduced, both the success rate of the learning session and the recognition rate decrease. Simulations of learning sessions with different number of epochs (200, 50, 10, 8, and 5) are carried out. With a number of epochs of 8, 2 learning sessions out of 3 failed, and with 5 epochs the SNN can never perform a successful learning. After concluding a successful learning session, the same test images (see Supplementary Figure 2) are shown to the SNN during recognition. The recognition rate decreases from 88.22% (200 epochs) to 82.61% (50 epochs), 75.29% (10 epochs), and 72.03% (8 epochs). This means that, when a limited number of epochs is performed, the synapses may be insufficiently depressed and during recognition they may conflict with the potentiated ones, thus resulting in incorrect recognition.

## 4. Discussion

In Section 3 a filamentary HfO_2_ memristor featuring analog behavior is presented. The proposed device is able to emulate both long-term plasticity and STDP learning rule. Moreover, a simple fully-connected SNN which takes advantage of the memristor plastic behavior and which uses an associative unsupervised STDP-based learning protocol is simulated. After a training session the network is able to recognize five characters, even when the images displayed are incomplete or noisy. It should be mentioned that non-ideal elements, such as parasitic or jitter, are deliberately not considered in the proposed network, because the performed investigation focuses on the basic principles of the network with analog memristor, where a study at a high level of abstraction is mandatory before considering practical implementations.

We demonstrate long-term functional plasticity with two different spiking algorithms, which have been already used in the literature (Park et al., 2013; Yu et al., 2013a; Li et al., 2014; Zhao et al., 2014) to emulate plasticity. The two algorithms allow an investigation on the device behavior as a function of the voltage amplitude (Figure 2A) and on its integrative response when stimulated by identical spikes (Figure 2B). An algorithm that modulates the spike voltage applied to the device is not easy to be implemented in a system. Indeed, dedicated read-out and variable voltage biasing circuits are required. On the other hand, the voltage on the memristor in a system could be modulated through superimposition of long spikes, as proposed several times in literature (Serrano-Gotarredona et al., 2013; Saïghi et al., 2015). This method allows the neuron to always fire the same spike and let the delay times between spike determine the actual voltage on the device.

**Figure 7:**
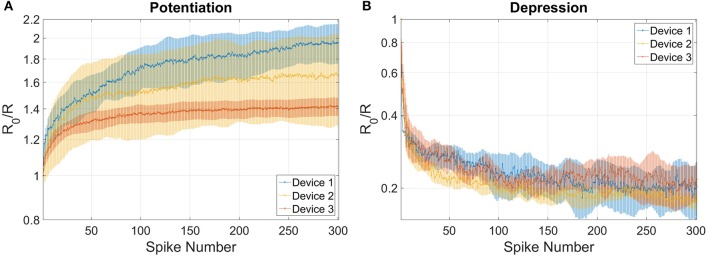
**Variability in the behavior of 3 different devices for (A) potentiation and (B) depression when stimulated by trains of 300 identical spikes**. Potentiation: voltage amplitude −0.55 V, time width 25 μs. Depression: voltage amplitude 0.75 V, time width 20 μs. Symbols indicate the mean value of 10 repetitions of the same train of spikes and the shaded area indicates the standard deviation.

**Figure 8:**
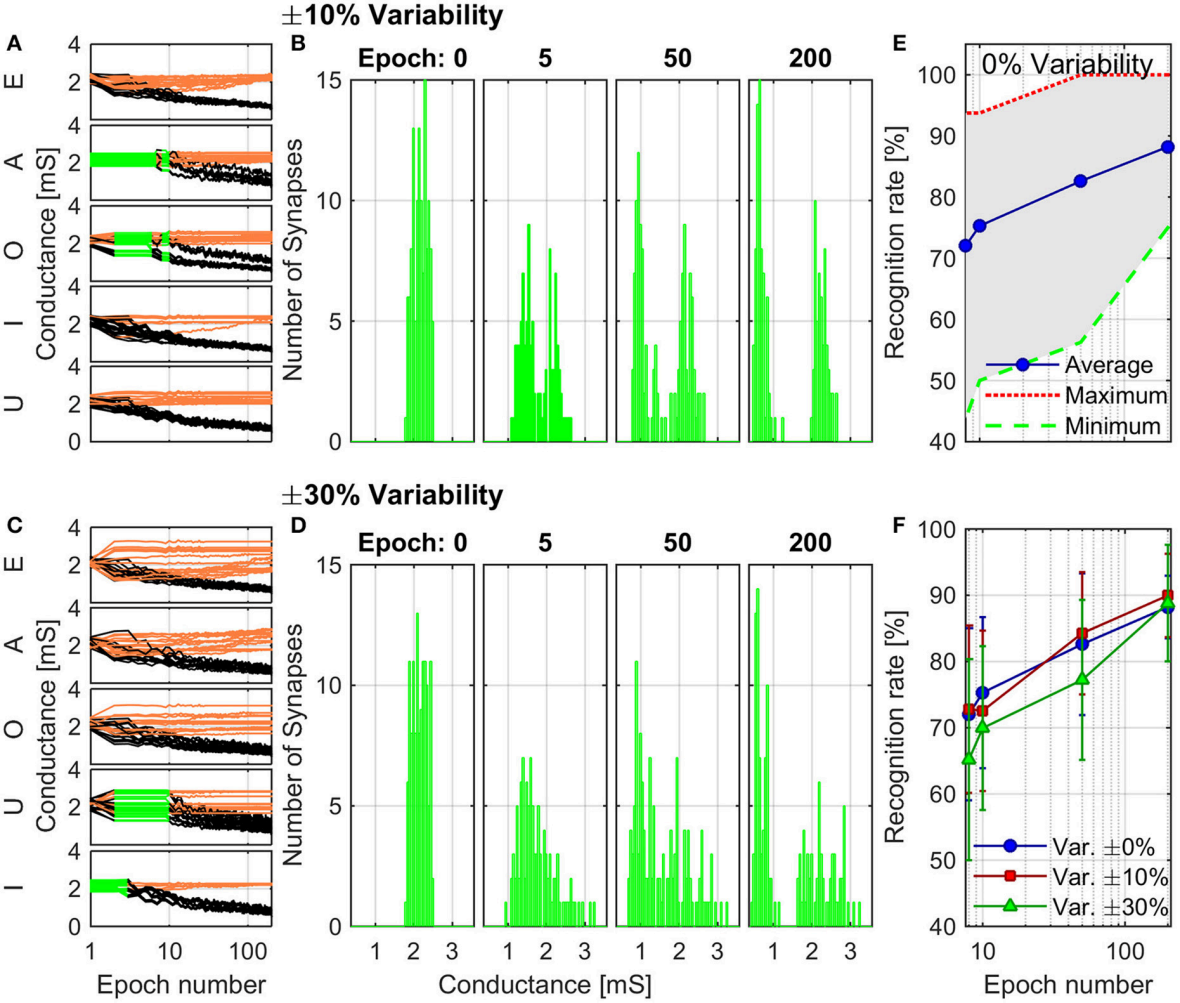
**Simulation of the training session including ±10% (A,B) and ±30% (C,D) of variability in synaptic behavior. (A,C)** Detailed synaptic weight evolution during training session of all characters. Black lines represent the synapses which are being depressed during the session and orange lines the ones potentiated. Green lines indicate that the neuron did not fire in the corresponding epoch. **(B,D)** Distribution of the synaptic weights during the training session. **(E)** Recognition rate as a function of the number of epochs in the learning session. The blue circles represent the average recognition rate from 100 simulations where device-to-device variability is not taken into account. The red dotted line and the green dashed one indicate the best and worst results obtained in the simulations, respectively, whereas the other results lie in the shaded gray area. **(F)** Average recognition rate of 100 simulations as a function of the number of epochs in the learning session with device-to-device variability of 0% (blue circles), ±10% (red squares), and ±30% (green triangles). Error bars show the standard deviation of the results.

The combined results of the measurements shown in Figure 2 are used to engineer the shape of pre- and post-spikes used to emulate homosynaptic plasticity and to conceive a biologically plausible STDP curve (Figures 4C,D), which takes advantage of both the relative timing between the two spikes (Δt) and the plasticity given by spike pair repetition. It should be noted that, though analog changes can be obtained around the previously found thresholds for potentiation and depression, the device can be operated in an analog fashion in a range of voltage of some hundreds of mV (Figure 4). From Figures 4A,B, it can be observed that voltages from 580 to 800 mV for depression and from −440 to −650 mV for potentiation allow a resistance evolution as a function of the repetition of identical spikes. In particular, Figures 4C,D show that the dynamic range decreases with the decreasing of the applied voltage, but resistance still gradually changes. In a network, it can be expected that different devices show analog transitions for a range of voltages whose end values (V_*min*_, V_*max*_) can be different from device to device, but in general a sub-range of voltages allowing analog resistance modulation is shared by many devices. A threshold difference in the devices (provided it is within few tens to one hundred mV) would not prevent analog behavior, as demonstrated in Figure 7, which shows the behavior of 3 different devices during potentiation (Figure 7A) and depression (Figure 7B) when stimulated by trains of 300 identical spikes. In both Figures 7A,B, the mean value of 10 repetitions of the same train of spikes is represented by symbols and the shaded area indicates the standard deviation of the measurements. It can be noted that potentiation suffers of major variability with respect to depression. Nevertheless, despite the device-to-device variability, all the devices show an analog behavior in both operations. In addition, different resistance evolutions due to different device thresholds are compensated in SNNs by the high parallelism of the architecture itself which enhances the network tolerance to device variability (Yu et al., 2013a). In this respect, the performance of the presented SNN against variability is tested adding ±10% (Figures 8A,B) and ±30% (Figures 8C,D) device-to-device variability in the artificial synapses behavior, i.e., the look up table associated to each synapse has been multiplied by a random factor extracted between 0.9/1.1 and 0.7/1.3 respectively. Figure 8A summarizes the synaptic weight evolution during the training session of all the characters as a function of the epoch number when a variability of ±10% is set. Each graph shows the weight evolution of the group of synapses contributing to the firing of a specific post-neuron. During learning, depression (black) and potentiation (orange) of synapses occur, but the weight evolution with and without variability (as in Figure 6B) is different, because in the former case for some presentation of the images to the network some groups of synapses are not updated (green lines for synapses connecting to post-neuron that is finally specialized to characters *A* and *O*). This is explained as follows. In the examples reported in Figure 8, first, *O* is presented and post-neuron *O* (meaning post-neuron that finally specializes to recognize *O*) starts firing and updating its associated synapses in the first epoch. On the other hand, variability causes that the weight are adjusted in such a way that from epoch 2 to 6, a different post-neuron fires and synaptic weights associated to post-neuron *O* are frozen. Then, specialization proceeds with one post-neuron specializing for only one character. The success of the learning session demonstrates the robustness of the network against device-to-device variability, in accordance with Yu et al. (2015), provided analog behavior holds in each device. Figure 8B shows the weight distribution of the synaptic matrix during training. Increasing the number of epochs, the initial synaptic weight distribution tends to separate in two groups, one for depressed synapses and one for potentiated synapses, as it happens also in Figure 6D. However, in the case of Figure 8B, the two distribution are wider than in the case where no variability factor is considered. The same above observations are valid also when variability is increased to ±30%, as shown in Figures 8C,D. Indeed, also Figure 8C shows, in the bottom two graphs, some epochs where the synaptic weight is not updated. Moreover, considering the ±30% variability test, the final distribution of the synaptic weights is larger than the one achieved for ±10% variability (17% larger for depression and 136% larger for potentiation). In this respect, it is worth analyzing the recognition rate of the test set shown in Supplementary Figure 2, as a function of the number of epochs carried out during learning. Figure 8E shows the recognition rate (blue circles) as a function of the number of epochs in a SNN neglecting device variability. Each circle is the average recognition rate over 100 simulations (i.e., 100 learning sessions each starting with a different initial configuration of the synaptic weights) and the results of each simulation lie in the gray shaded area delimited by the best simulation result (dotted red line) and the worst one (dashed green line). The increase of the number of epochs during learning improves the average recognition rate and decreases the spread of the results. Indeed, the recognition rate varies between 43.75 and 93.75% at 8 learning epochs whereas it varies between 75 and 100% at 200 learning epochs. As already mentioned in Section 3.3, the recognition rate is closely related to the distribution of the synaptic weights at the end of the training session. The nearer the distributions of the potentiated and depressed synapses, the lower the recognition rate. As a consequence, the increase of the number of learning epochs contributes to enhance the separation of the two above-mentioned distributions and, therefore, to improve the recognition rate. It is interesting to note that in this respect the impact of device-to-device variability is almost negligible. Indeed, we performed the same recognition tests with the same methodology also in case of SNNs with ±10 and ±30% device-to-device variability. Figure 8F shows the average recognition rate as a function of the epochs during learning in case of 0% (blue circles), ±10% (red squares), and ±30% (green triangles) device-to-device variability. The vertical bars indicate the standard deviation σ. The same increasing trend can be noted for all the curves regardless of the variability. In accordance with Figure 8E, in each curve also σ decreases with increasing number of epochs, but the value of σ for each number of epochs during learning increases with increasing variability. On the other hand, the network proves to be robust also for variability up to ±30%. The network robustness lies in the gradual synaptic weight update. Indeed for every post-neuron spike, the weight is adjusted by a small amount. If an erroneous spiking (like the one of a post-neuron responding to two different characters) occurs, the weight change is small enough that the following epochs can recover the error.

Given the observations above, we would like to stress that it is fundamental in deterministic networks to have analog synapses even though, as in the proposed SNN, the images shown are only black and white. Indeed, in a system with deterministic neurons, as in the proposed one, binary deterministic memristors would lead to fast learning (only few epochs would be necessary to complete the training session), but also to fast forgetting (Fusi and Abbott, 2007). Indeed, if a noisy image were shown to a trained SNN employing binary synapses, the network would classify that image and, therefore, would adjust the synaptic matrix also according to the pixel which is not representative for that image, disrupting learning. In the case of analog synapses, the same permanent and significant change leading to failure would result only if the same noisy image were shown to the network for several epochs, which is statistically improbable.

In the presented SNN, using two fixed delay times (one for potentiation and one for depression) in the STDP is sufficient as a proof-of-concept. In this respect, two values are selected (Δt = 300 μs and Δt = −50 μs) which are coherent with a post-neuron firing as a consequence of the stimulation by the pre-neuron (synapses potentiation for Δt = 300 μs) rather than with a pre-neuron firing because of the stimulation by the activated post-neurons in case of synaptic depression (Δt = −50 μs). On the other hand, a network exploiting also the possibility of variable delay times between pre- and post-spikes, would allow increasing the available resistance states, therefore, improving the network robustness even further. As an example, in the case of input-specific associative learning rules for pattern recognition, the possibility to combine different parameters (Δt and spike pair repetition) to achieve various resistive states with different evolution histories offers a further degree of freedom. Indeed, a possible application could be in networks where images have different colors o shades of gray, which can be linked to different delay times. In this case, at the end of a learning session with a certain number of epochs, the weight distribution of the synaptic matrix would give an indication of the common features of the various images presented to the network. More specifically, the more a group of synapses is potentiated, the more they are stimulated, i.e., the potentiated group identifies a common feature in the set of displayed images.

## 5. Conclusion

In summary, a thorough analysis of the synaptic features of the proposed oxide-based memristor is carried out. Initially, the device ability to emulate long-term functional potentiation and depression is proved upon stimulation with spikes with increasing amplitude (stair-case like) and trains of identical spikes. These experiments show that the memristor has an analog behavior in tuning its resistance and it can reach a dynamic range up to one order of magnitude depending on the spiking algorithm employed. Then, homosynaptic plasticity is tested through STDP experiments, which demonstrates the device biological-like behavior when subjected to synaptic activity. Finally, the possibility of developing deterministic networks using unsupervised learning is investigated. A subset of the STDP collected data is used to simulate a simple fully-connected SNN featuring an associative unsupervised STDP-based learning protocol. The network is able, after a training session, to recognize the five characters, also when partially incomplete or noisy letters are displayed. Therefore, the SNN proves that the proposed memristor can be used to emulate the functionality of an artificial synapse in future neuromorphic architectures with deterministic neurons, and analog memristive synapses, and making use of unsupervised learning for real-time applications.

## Author contributions

EC, SB, and SS conceived the experiments and wrote the manuscript. SB and SS developed the memristor device. EC and SB collected the data on synaptic plasticity, in collaboration with AS. EC and SB performed the STDP experiments. EC and SB developed the SNN, in collaboration with AS. All authors discussed the results and contributed to manuscript preparation.

## Funding

The work has been partially supported by the FP7 European project RAMP (grant agreement n. 612058).

### Conflict of interest statement

The authors declare that the research was conducted in the absence of any commercial or financial relationships that could be construed as a potential conflict of interest. The reviewer SJ and handling Editor declared their shared affiliation, and the handling Editor states that the process nevertheless met the standards of a fair and objective review.
