# Supplementary material for: Analog Memristive Synapse in Spiking Networks Implementing Unsupervised Learning
Source: Front Neurosci. 2016 Oct 25;10:482. doi: 10.3389/fnins.2016.00482 (PMC5078263; doi:10.3389/fnins.2016.00482)
Supplement: Supplementary file 3 [file DataSheet1.PDF]

Supplementary Material: Analog memristive synapse in spiking networks implementing unsupervised learning

Erika Covi*, Stefano Brivio, Alexander Serb, Themis Prodromakis, Marco Fanciulli, and Sabina Spiga*

*Correspondence:
Erika Covi
erika.covi@mdm.imm.cnr.it

Sabina Spiga: sabina.spiga@mdm.imm.cnr.it

1 SUPPLEMENTARY DATA

The Graphic User Interface (GUI) developed to enhance the usability of the simulated Spiking Neural Network (SNN) is shown in Fig. 1. On the top field, it is possible to indicate the path to save a simulation or a video. The matrix of 25 white/black pixels represents the input image. A character can be chosen from the drop down menu on the right. Otherwise, an image can be inserted by manually clicking the buttons of the input matrix. Below the drop down menu, there is a field where the number of learning epochs can be specified.

After a character is displayed in the input matrix, button *Start learning* should be clicked to to start training the network to recognize the selected character. Once the training is completed, recognition test can be performed by first inserting the desired pattern in the input matrix, then clicking *Start recognition*. Button *Graphs* shows the graphs describing the evolution of the synaptic weights during the learning session and the corresponding weight histograms. Button *Save simulation* saves current simulation, whereas button *Load simulation* loads the last simulation saved. If one wants to perform a new simulation, he should click *New simulation* button.

In the bottom part of the window, videos showing the evolution of the synaptic weights for one or all the characters, as in the videos provided in the Supplementary Material of this manuscript, can be prepared setting the number of epochs to be recorded in the video and the speed (frames per seconds) of the video, then pressing the *Start making video* button.

Fig. 2 shows the set of testing images displayed to the SNN during recognition. 9 images with missing pixels (gray pixels in the figure) and 7 images with noise (red pixels in the figure) are shown to the network. The test set was chosen in order to enhance the competitiveness among post-neurons. At first, the black pixels in common between the letters were analyzed. Fig. 3 shows how many letters use each pixel, e.g., pixel 1 is used for two characters (*E* and *U*), pixel 3 is used for four characters (*A*, *E*, *I*, and *O*), pixel 8 is used only by one character (*O*), and so on. When testing the SNN with incomplete images, the missing pixels were chosen among the ones common to less characters. This way, the signal contribution to the post-neuron specialized for the letter shown is decreased and, consequently, the competitiveness between post-neurons is stimulated. Similarly, the noisy pixels were chosen among the ones common to more

characters in order to increase the competitiveness between post-neurons and evaluate the robustness of the SNN.

In Table 1 the results of the recognition tests when learning session is made up of 200, 50, 10, and 8 epochs are summarized.

2 SUPPLEMENTARY TABLES AND FIGURES

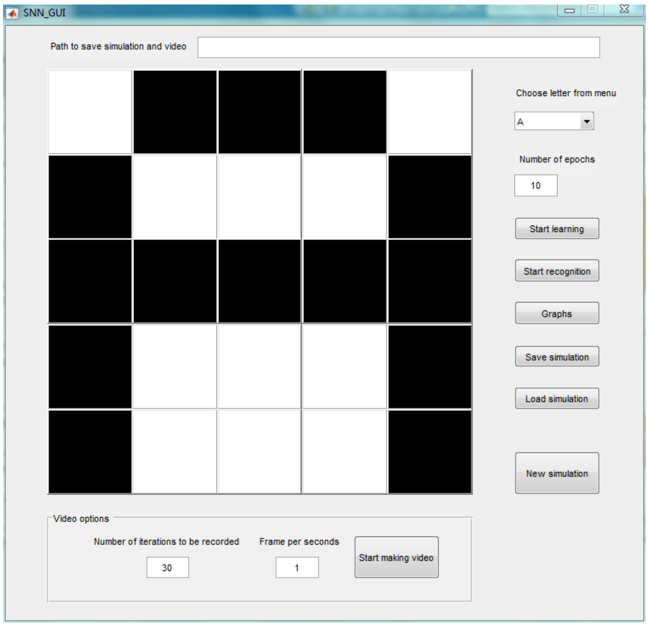

Supplementary Figure 1. GUI of the developed SNN.

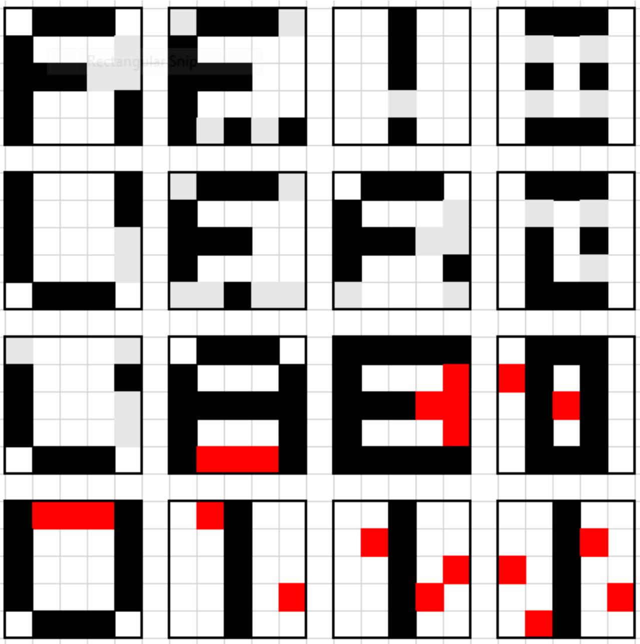

Supplementary Figure 2. Test images shown during recognition. For ease of understanding, missing pixels are colored in gray and noise pixels in red.

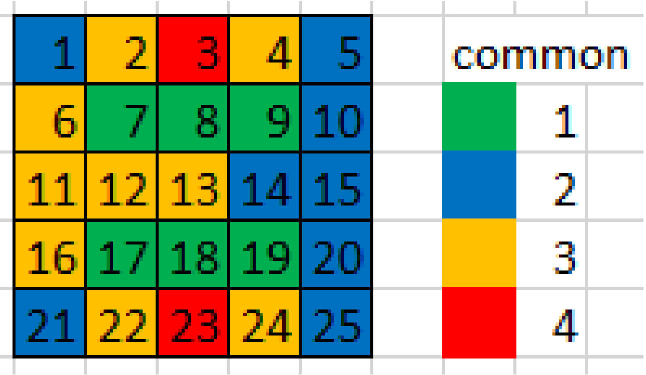

Supplementary Figure 3. Pixels used by each character.

Table 1. Recognition tests - results when the learning session is made up of 200, 50, 10, and 8 epochs

Test number	Character shown	Missing pixels	Noisy pixels	Recognized character			
				200 epochs	50 epochs	10 epochs	8 epochs
1	A	3 out of 14		A	A	A	A
2	E	4 out of 15		E	E	E	**A**
3	I	1 out of 5		I	I	I	**E**
4	O	4 out of 12		O	O	O	**E**
5	U	2 out of 11		U	**E**	**E**	**E**
6	E	5 out of 15		E	E	E	**A**
7	A	5 out of 14		A	A	A	A
8	O	3 out of 12		O	O	O	O
9	U	4 out of 11		U	U	**E**	**E**
10	A		3	A	A	A	A
11	E		4	E	E	E	**A**
12	O		2	O	O	O	O
13	U		3	**E**	**E**	**E**	**E**
14	I		2	I	I	I	I
15	I		3	I	I	I	I
16	I		4	I	I	I	I
